# A Mixed-Methods Evaluation of CARE (Cancer and Rehabilitation Exercise): A Physical Activity and Health Intervention, Delivered in a Community Football Trust

**DOI:** 10.3390/ijerph18063327

**Published:** 2021-03-23

**Authors:** Zoe Rutherford, Stephen Zwolinsky, Nicky Kime, Andy Pringle

**Affiliations:** 1 School of Public Health, Faculty of Medicine, The University of Queensland, Brisbane, QLD 4006, Australia; 2 Policy and Epidemiology Group, Queensland Centre for Mental Health Research, Brisbane, QLD 4072, Australia; 3 West Yorkshire & Harrogate Cancer Alliance, White Rose House, West Parade, Wakefield WF1 1LT, UK; s.zwolinsky@nhs.net; 4 Bradford Institute for Health Research, Temple Bank House, Bradford Royal Infirmary, Bradford BD9 6RJ, UK; Nicola.Kime@bthft.nhs.uk; 5 Department of Sport, Outdoor and Exercise Science, School of Human Sciences & Human Sciences Research Centre, University of Derby, Kedleston Road, Derby DE22 1GB, UK; A.Pringle@derby.ac.uk

**Keywords:** cancer, exercise, physical activity, rehabilitation, intervention, football in the community, behaviour change, RE-AIM

## Abstract

With increasing cancer survivorship has come an increased necessity to support people living with cancer (PLWC) to have a good quality of life including being physically active. Using mixed methods, the current study aimed to use the RE-AIM evaluation framework (Reach, Effectiveness, Adoption, Implementation and Maintenance) to determine how the football community trust delivered CARE (Cancer and Rehabilitation Exercise) intervention was able to increase participants’ physical activity in order to improve their quality of life and regain physiological and psychological function. Quantitative outcome data were collected at baseline, 3 and 6 months using the Cancer Physical Activity Standard Evaluation Framework questionnaire. Semi-structured focus groups (*n* = 5) captured participants’ (*n* = 40) lived experience of the reach, effectiveness, adoption, implementation, and maintenance of CARE. Questionnaire data were analysed using repeated measures ANOVAs and qualitative data were thematically analysed. Following diagnosis, CARE was successful in providing participants with a unique and accessible opportunity to become or restart physically activity, by providing a local, socially supportive, and inclusive environment. This resulted in significant increases in physical activity (*F*_(1.58, 23)_ = 5.98, *p* = 0.009), quality of life (QoL) (*F*_(2,36)_ = 13.12, *p* = 0.000) and significant reductions in fatigue (*F*_(1.57,31)_ = 11.19, *p* = 0.000) over 6 months. Participants also reported becoming more active, recovering physical function, regaining independence, and enhanced psychological well-being as a result of attending CARE. Key design features of CARE were also identified across RE-AIM. CARE, a football community trust delivered physical activity intervention was successful in significantly improving participants’ QoL and in regaining the physical and psychological functioning of people living with cancer. Results suggest that maintaining engagement in CARE for 6 months and beyond can support people to maintain these changes. Engaging in robust evaluations such as this can help organizations to successfully secure future funding for their programs.

## 1. Introduction

Each year, approximately 365,000 people are diagnosed with cancer in the UK [1]. This number is set to increase by circa 2% each year due to a myriad of socio-demographic and behavioural influences [2]. Nevertheless, improvements in screening, diagnosis and treatment mean that the number of people living longer after a cancer diagnosis is likely to increase by 3% each year going forward [3]. While this news is encouraging, it does however mean that many people are also living with the negative and debilitating side effects of some treatment, requiring additional health and social care resources [4]. With increasing cancer survivorship has come an increased necessity to support people to have a good quality of life (QoL) [5] including being physically active [6].

Physical activity and exercise have been shown to improve the negative physiological and psychosocial consequences of cancer treatment that exist for many, such as fatigue and health related QoL [7,8,9] anxiety and depression [10], and in the case of breast and colon cancer, a reduced risk of recurrence [11,12]. Those who have higher pre-diagnosis and post-diagnosis levels of physical activity have also been associated with improved survival. Furthermore, people who have cancer are significantly more likely to be inactive than those without cancer [13], and are more sedentary. Despite this, less than half of people living with cancer (PLWC) are sufficiently active to benefit their health (e.g., 18% of UK men and women [14]; 19% of women with ovarian cancer [15]; 30% mixed cancer [16]; 45% of those with breast cancer [17]), meaning that there is much need for the promotion of physical activity following a cancer diagnosis [18].

More broadly, physical inactivity (or low physical activity) has been referred to as a global pandemic [19] and bold societal and governmental action has been recommended, including the use of innovative approaches and settings for health improvement [20,21,22]. In 2006, Fédération Internationale de Football Association (FIFA) recognised the unique role that football could play in the promotion of exercise and healthy behaviours, which in turn could reduce the burden from communicable and non-communicable diseases in many countries. Football club community trusts are the charitable arm of professional clubs across the English Premier and Football Leagues [23] and are increasingly recognized as an important ingredient of the health improvement infrastructure [24] and are highlighted in national policy [25]. This mechanism of health promotion has been found to be effective in improving cardiovascular health and fitness in men and women [26]; weight management in men [27,28,29] and women [30,31]; and in improving physical activity and social inclusion of men with mental health problems [32]. Indeed, in some instances, successful interventions [27] have been rolled out nationwide with significant financial support. Despite this, there have been few studies [33,34] that have evaluated the effectiveness of football club community trust led programs offering physical activity and behaviour change interventions for cancer patients (both men and women) and to the best of our knowledge none in the UK.

There is a well-documented need for accessible programs to promote exercise participation among adults during and after cancer treatment [35]. Furthermore, Ax et al. [36] report that in order to develop and implement relevant rehabilitation programs, it is ‘important to increase knowledge about how PLWC experience the effects of physical activity on their functioning in daily life and to examine the role it plays in their recovery’. The Cancer And Rehabilitation Exercise (CARE) program is a football community trust delivered behaviour change intervention, in the City of Nottingham (England, UK), which aims to support PLWC to become more active, in order to recover physical function, reduce the risk of developing other long-term conditions, regain independence and enhance psychological well-being. The RE-AIM framework [37] has been used to help evaluate health interventions, featuring in 2800 publications, including interventions delivered by football ball community trusts and foundations, commonly referred to as football-led health improvement programs [38]. The aim of this study was to use the RE-AIM framework as a guide to evaluate the Reach, Effectiveness, Adoption, Implementation and Maintenance of CARE on participants’ questionnaire measured physical activity, fatigue, health related QoL, confidence in daily living; and qualitatively through their lived experiences, to inform the future program development and commissioning of the service.

## 2. Materials and Methods

### 2.1. Study Design

This mixed-methods retrospective study was conducted using the RE-AIM framework as a guide to evaluate the Reach, Effectiveness, Adoption, Implementation and Maintenance of CARE. Table 1 describes each element of RE-AIM in the context of the study and the quantitative and qualitative data collected.

### 2.2. Intervention Context and Setting

**Figure 1:**
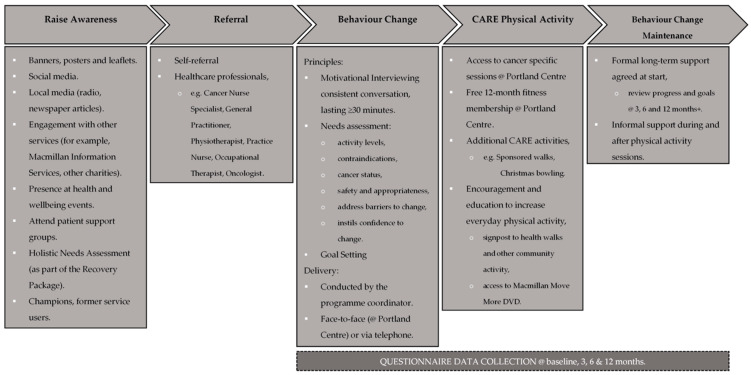
Overview of the key components of the Macmillan Physical Activity Behaviour Change Care Pathway applied to CARE.

Notts County Foundation (NCF; formally known as Notts County FC Football in the Community) are an established football community trust organisation with a proven track record in delivering successful community-based health improvement interventions [31]. In response to local need, NCF, in partnership with Macmillan Cancer Support and informed by the Macmillan Physical Activity Behaviour Change Care Pathway [39]; designed, funded and established CARE in March 2015, for PLWC across Nottinghamshire, England. Service users were referred onto CARE via various primary care providers (e.g., Cancer Nurse Specialists, General Practitioners, Occupational Health Consultants, Oncologists, Physiotherapists, Practice Nurses, Psychologists, Radiographers), or via self-referral. The CARE program (see Figure 1 for an overview of the key components) was overseen by an appropriately qualified (L4 Cancer & Exercise Rehabilitation https://pdphub.com/event-pro/cancer-rehabilitation-foundation-training/ (accessed on 27 October 2019)) programme coordinator and delivered from the Portland Centre; a NCF run leisure centre situated in the Meadows area in the City of Nottingham. For the duration of the evaluation period, the CARE program was free for service users.

On referral onto the program, the programme coordinator conducted an induction interview (in person or via telephone) to discuss the individual’s needs, motivations, and barriers to taking part in physical activity and assess their readiness to participate. Thereafter, the programme coordinator supported service users to set achievable goals and discussed how they would achieve those through the program, by way of a motivational interviewing (MI) consistent conversation. All service users were required to complete an evaluation questionnaire, developed from the Cancer Physical Activity Standard Evaluation Framework (CaPASEF) [39]. Follow-up sessions with the programme coordinator took place with each service user at 12 weeks, 6 and 12 months to review participants’ goals and to complete the evaluation questionnaire [40].

Since its conception in March 2015, the structure of the CARE program developed from being delivered in blocks of 12 weekly sessions lasting one and a half hours each, with three sessions being delivered each week: One targeting women only, one targeting men only and a mixed session. As a result of ongoing service evaluations conducted by NCF and further funding from Macmillan, CARE sessions were offered as a rolling program, meaning service users were able to attend CARE beyond 12 weeks and were able to start whenever they were ready (i.e., avoiding the need for a waiting list). Although CARE was delivered by a football foundation which are an important public health deliverer, CARE has little to do with the game of football but does reflect the diverse range of public health and social inclusion work football foundations and trusts undertake. Further, a review of health improvement work delivered by 72 English Football League Trusts [41] and also Spanish Football clubs identified no specific programmes for cancer survivorship [42], thus reflecting the novelty of this programme as delivered in English football. CARE also deployed a range of physical activity modes. each session was delivered by NCF coaches and included a warm-up (incl. stretching and light cardiovascular work), a main activity chosen from an extensive menu the week before (e.g., gym-based exercises, circuits, soccercise, Zumba, yoga, badminton, and indoor tennis) and a cool-down activity. In order to support CARE service users to be physically active outside of the organised sessions and develop long term physical activity behaviours, participants were provided a free membership to the Portland Centre (for 12 months), access to Macmillan resources (healthy eating recipe books, booklets on physical activity and cancer) and signposting onto other activities in Nottingham/Nottinghamshire (e.g., health walks, walking netball, and Nifty 50s).

### 2.3. Participants and Procedures

The inclusion criteria for CARE, service users had to be (1) be aged >18 years and (2) have or have had a cancer diagnosis. Due to these broad criteria, we were unable to determine the total number of eligible participants and therefore the proportion of the PLWC taking part in CARE in terms of overall Reach, and audit data were used to determine the number of PLWC reached.

Prior to the baseline questionnaire completion and focus groups, service users were asked to provide their consent for their data to be used for research purposes via a project participant information sheet and consent form. Only the data of those who returned informed consent forms were included within this study. Ethical approval was obtained through Leeds Beckett University’s Local Research Ethics Committee for both quantitative and qualitative parts of the evaluation (ID: 45555).

#### Evaluation Questionnaire

To satisfy the funding requirements of the CARE program, all CARE service users were required to complete the evaluation questionnaire, determined through the CaPASEF. The CaPASEF was commissioned by Macmillan and developed by researchers in order to standardise evaluations of physical activity interventions for PLWC [40]. The evaluation questionnaire contains several validated outcome measures used to examine changes over time and these have been used to quantitively reflect the effectiveness of CARE. A brief description of the demographic items and the physical activity and health outcome measures forming the overall questionnaire are as follows:

*Demographic information:* Age, gender, ethnicity, relationship status, socio-economic status (SES; proxy via education), disability or illness, cancer diagnosis (type, treatment, and status). These data are presented as means (± *SD*), to describe the PLWC the CARE programme reached.

*Total physical activity minutes* (Scottish Physical Activity Questionnaire (SPAQ) [43]): This measure asks participants how many minutes they spent in the previous week undertaking different activities such as walking and housework. For this study, all the minutes were summed to obtain a total amount of minutes for the week and the higher the number of minutes, the more activity is undertaken.

*Fatigue* (Functional Assessment of Chronic Illness Therapy—Fatigue (FACIT-F) [44]): This is a 13-item questionnaire that assesses self-reported tiredness, weakness, and difficulty conducting usual activities (impact) due to fatigue. Each question has a scale of 0 to 4 where 0 indicates “not fatigued at all” and 4 indicates “very much fatigued”. Participants are asked to score where they felt they were on that scale for each question over the past 7 days. All responses to the 13 questions are summed to obtain a single overall score for fatigue (ranges from 0–52), with a higher score indicating higher levels of fatigue.

*Health Related Quality of Life* (EQ-5D [45]): This questionnaire asks two different types of questions: In the first part of the questionnaire participants are asked to describe the level of problems they experience in five areas: (1) Mobility, (2) self-care, (3) usual activities, (4) pain/discomfort and (5) anxiety/depression. Each question is divided into three degrees of severity: No problem, some problems and major problems. Each of these is scored between 0 and 1 where a value of 0 indicates the worst possible state of health and a value of 1 indicates full health. The second part is a 20 cm visual ruler running from 0 to 100 on which the participant reports their current health condition. A score of 0 indicates the worst imaginable health and a score of 100 indicates the best imaginable health.

*Confidence in daily life* (General Self-Efficacy Scale [46]): The questionnaire consists of ten items with statements such as “I can always manage to solve difficult problems if I try hard enough” and “When I am confronted with a problem, I can usually find several solutions”. Response categories range from 1 to 4 where 1 indicates “not at all true” and 4 indicates “exactly true”. A total score is calculated by totalling all responses, which range from 10 to 40 points, with higher scores indicating higher levels of general confidence.

### 2.4. Focus Groups

During January and February 2018, service users attending the CARE were invited to take part in semi-structured focus groups [FG] to explore service users’ experiences of the CARE program and its impact, using the RE-AIM framework as a theoretical framework to guide the evaluation. The interview schedule for the focus groups were developed from our previous involvement in the evaluation of football-led health improvement interventions [24,38,47,48,49,50]. The question areas can be loosely aligned with the components of RE-AIM. Rather than viewing the components of RE-AIM as being tightly defined with immovable boundaries, the reality is that in some instances the components permeate. For example, Implementation does exist in a vacuum, it permeates across the other components of Reach, Adoption and/or Maintenance. This knowledge was developed from our previous work [50,51]. Prior to their application, the schedules were piloted, and the FG leader practiced their interview techniques. Following each FG the interviewers met to reflect on how each group had worked and discussed any key learning for future iteration.

Focus groups and interviews have been used previously to investigate participant experiences of attending football-led health improvement interventions reflecting both the implementation component of RE-AIM [37] and the key characteristics such as the Premier League Men’s health Evaluation and the Fit Red’s men’s health evaluation [32,52]. They can be an inclusive, efficient, and convenient method to capture information on participant experiences of engaging with interventions. A total of four focus groups were undertaken with participants engaging CARE. At the point of data collection, all service users of the CARE program were provided with the study information sheet and asked to return the informed consent forms if they were happy to take part in a focus group. Only those providing informed consent were eligible to take part. The size of groups ranged between 4–7 participants and were gender specific (males and females), to make the focus groups accessible for participants to engage and share their accounts, some of which may be sensitive in nature. The focus groups took place after the programmed sessions and were held in a meeting room at the Portland Centre. Focus groups lasted 45–60 min and were digitally recorded.

Prior to inviting participants to engage in the focus group, the researchers attended a series of social events such as the Christmas bowling event and planned CARE activities to build up rapport with potential volunteers. This approach has been adopted elsewhere in research studies investigating the impact of football-led health improvement programs [32,52]. This meant that on the day of the focus groups, participants recognised the researchers and that a previous connection had already been established [52] and thus contributed to building capacity for research recommended in the literature [48]. Further, previous relationships between participants helped facilitate participant engagement and interaction in the focus groups.

### 2.5. Data Reduction and Analysis

#### 2.5.1. Questionnaire Data

All completed questionnaires were passed to the research team in line with ethical procedures upon completion of the evaluation period and data were inputted into a password protected Excel spreadsheet anonymously. Data were subsequently transferred to SPSS (IBM SPSS Statistics for Windows, Version 27.0. IBM Corporation, Armonk, NY, USA.) for cleaning and analyses. In line with Moreton et al. [39], a “completers only” approach to analysing the outcome data was deployed. While the CARE program did not have a clear completion point, for the purposes of this study we identify completion in terms the three follow-up data collection points. Completers are therefore defined as someone who has provided useable questionnaire data at baseline, 3, 6 and 12 months. However, while this is not perfect—not everyone who remains engaged with CARE completes such follow-up questionnaires, it is the only indication we have available. This approach is also in line with that of Moreton et al. [39] and provides the best way of determining changes in physical activity and health related quality of life changes over time, given the constraints of the data collection method. Total SPAQ measured physical activity were calculated and for those days where total minutes exceeded 1400, these were removed as unrealistic [38]. Descriptive statistics were performed to describe the population and changes between baseline and 3 months using paired samples t-tests, with significance set to *p* < 0.05.

#### 2.5.2. Qualitative Data

Following the focus groups and interview, the recorded audio files were transcribed verbatim by a professional transcriber. As the interview schedules were structured to follow the RE-AIM framework, Braun and Clarke’s [53] six stages of thematic analysis were deployed to determine the sub-themes of the a priori reach, effectiveness, adoption, implementation and maintenance themes. For the interview data and following transcription and immersion of the interview transcripts to saturation, coding identified interesting features in the data, and these were grouped into coherent themes using the RE-AIM framework components. A visual map was developed by hand to show the themes and their relationship. Two researchers’ (A.P. and N.K.) met to refine the specifics of the themes and to generate clear definitions and names for them, which were then shared with the lead and co-author (Z.H.R. and S.Z.). This approach has commonly been used in the investigation of football-led health improvement programs [32,54].

## 3. Results

In order to best reflect the aims of the study, the results are presented under the RE-AIM framework.

### 3.1. Reach

Between March 2015 and June 2018, 346 people took part in CARE, as audited by NCF (*n*= 238 female, *n* = 108 male). At the point of evaluation, and despite all completers providing consent, questionnaire data were only available for *n* = 170 participants at baseline, *n* = 101 at 12 weeks, *n* = 37 at 6 months and *n* = 14 at 12 months, with data cleaning further reducing the sample sizes for different variables. Due to the small sample sizes at 6 and 12-months from which we can draw any meaningful conclusion, these time points were excluded from statistical analysis. Further, five focus groups took place with all those who provided consent (males *n* = 23, females *n* = 17). Table 2 shows the demographic profile and characteristics of the participants of the evaluation at baseline.

Table 3 shows the cancer type, status, and treatment of CARE participants at baseline. For females, most participants were diagnosed with breast cancer (77%), whereas most males were diagnosed with prostate cancer (72%). Regarding participants’ current cancer status, 55% were in remission or cancer free and of those who were managing their cancer, 20% were stable. Important to note is that data show that participants were at differing stages of their treatment, with 48% of participants reporting that their treatment had been effective, 23% were currently in treatment, with the remaining participants awaiting treatment or under surveillance.

### 3.2. Effectiveness

The results of the paired samples t-tests conducted on the CaPASEF questionnaire data are presented in Table 4.

#### 3.2.1. Physical Activity

Following data cleaning, only 68 participants were included in the analysis. At baseline (Table 4), the participants of CARE were physically active for an average of 63 min per day. Results show that there was a significant increase in physical activity minutes over time (*t*_(67)_ = 3.84, *p* = 0.000).

#### 3.2.2. Fatigue

Participants displayed a significant reduction in total fatigue over time (*t*_(88)_ = 2.29, *p* = 0.024).

#### 3.2.3. Health Related Quality of Life

The results also demonstrate that health related QoL improved significantly over time (*t*_(100)_ = 7.89, *p* = 0.000). The number of participant responses by mobility, self-care, usual activities, pain/discomfort, and anxiety/depression from the EQ-5D are presented in Table 5 and show that participants demonstrated mixed but favourable changes across all dimensions.

#### 3.2.4. Confidence in Daily Life

Participants significantly increased their General Self Efficacy score over 3 months (*t*_(89)_ = 3.45, *p* = 0.001). The qualitative data (Table 6) show that participating in CARE had allowed several participants to increase their self-confidence and participants frequently reported regaining their sense of “me”. Alongside the significant positive changes in fatigue and QoL, the focus groups enabled participants to provide qualitative accounts of how CARE had affected their fitness, wellbeing, social connectedness and return to pre-diagnosis function (work and exercise), details of which are presented in Table 5.

### 3.3. Adoption

The themes relating to the profile of participants who engaged in CARE including their physical activity, health status, and reported barriers and facilitators [47] are presented in Table 7.

### 3.4. Implementation

Table 8 presents the key themes related to the key intervention design and delivery characteristics that participants reported as being influential in facilitating their adoption [50] of CARE. Human capital was important in facilitating adoption, including the skills and attributes of the CARE staff as well as positive support from fellow attendees. The development of self-management skills as also important in facilitating adoption.

### 3.5. Maintenance

Table 9 presents the themes related to the continued engagement of participants with CARE, in addition to aspects of the intervention that are key to the sustainability and future delivery of the programme and areas for improvement. It is also important to note that some of the sub-themes contained within the “Areas for Improvement” theme can also be linked to Reach.

## 4. Discussion

The present study used the RE-AIM framework to evaluate CARE, a football community trust delivered behaviour change intervention, which uses physical activity to improve the QoL and function of PLWC. The key findings were: (a) CARE was most successful in reaching women; and men, those from black, minority and ethnic (BME) and low socio-economic groups were under represented; (b) for those who adopted CARE, the intervention was effective in significantly improving the physical activity, fatigue, QoL and confidence in daily living of PLWC over a 3 month period; (c) the main reason why PLWC adopted CARE was that it was successful in alleviating people’s fears and anxieties about being active and engaging “mainstream” exercise provision; (d) the key features of CARE to reduce these barriers were the opportunity to exercise in a safe and supportive environment with staff and other PLWC who understood their situation; and (e) participants’ maintenance of physical activity is likely due to developing self-management skills and social networks through CARE. The findings are discussed in line with the RE-AIM framework to best represent the complexity of CARE and to inform future program development and commissioning of the service.

### 4.1. Reach

From the audited number of people who were recorded to have taken part in CARE during the evaluation period (i.e., adopters of care; *n* = 346), and despite the completion of the CaPASEF being a requirement of the programme, the proportion of CARE adopters included within the quantitative part of the evaluation (i.e., response rate) at baseline was 49%; 27% at 3 months and 11% at 6 months. It is unclear as to why there is such a difference in the reported number of people registered on the program and the number of completed questionnaires, but it may be that the participant burden of the questionnaires was too great for everyone to complete them. This may be especially true for participants who self-completed the questionnaire at 3, 6 and 12 months outside of the follow-up sessions. It may also be that this burden extends to staff in terms of data input and the storage of completed questionnaires. Indeed, in a report evaluating the Move More care pathway (the programme model that CARE is based on), authors reported that practitioners felt that the SPAQ, in particular, was challenging to complete and was the section of the questionnaire most likely to be returned incorrectly [39]. By reducing the number of questions or streamlining the questionnaires used, this may help to reduce participant burden and increase the data available for analysis. Additionally, by completing the questionnaire electronically and therefore automatically inputting the data, this may also help to improve the number of available data collected at each time point going forward, adding quantitatively to the reporting of effectiveness and maintenance.

In terms of representativeness, the population of CARE participants included within the evaluation were on average >55 years old, which is in-line with the proportion of PLWC in the UK, as age is a risk factor for most cancers, especially breast and prostate. Interestingly, most participants on CARE were women. The program is delivered by a football community trust, which have historically been designed to engage men. Research has highlighted the “reach of the club badge” and football has been shown to be a powerful instrument in connecting with people, including those unreached by statutory services [32,38], but in this case, few participants reported learning about CARE through NCF networks or were even interested in football. It is worthwhile recognising the potential reach of football and football clubs for different groups and circumstances; but contrary to the literature, there will be times when it is a less powerful mechanism for reaching intended audiences. Future research should explore the reasons why men living with cancer may be less engaged than women in interventions like these.

Despite around a quarter of the Nottingham population represented from BME groups in 2020 [55], there was little ethnic diversity within the CARE participants. Moreton et al. [38] reported that the reasons given by services with low rates of non-white British PLWC related to problems with access to the groups due to perceived cultural barriers or due to practical difficulties such as language barriers. According to the UK based Cancer Patient Experience Survey, black and minority ethnic cancer patients have poorer experiences of cancer services than their White British counterparts [56]. In a study exploring the reasons for this, Macmillan report that BME cancer patients tend to have greater emotional and psychological needs than White British people following cancer diagnosis and that they experience poor communication beyond their immediate physical needs with their GPs and hospital doctors, which also related to low levels of involvement about their care and treatment [56]. In order to engage more with this population, further research is needed to better understand how interventions can overcome these barriers and work with BME communities to develop networks. This is important, as people from BME backgrounds already report low levels of physical activity [57] and developing cancer is likely to further broaden the health inequalities associated with that.

While the area of Nottingham where this program is based is placed within the 10% most deprived in Nottingham City [58], the people attending the CARE program were mostly homeowners, with higher levels of education. This suggests that the program was less successful in engaging with lower socio-economic groups, which is of concern as those from lower socio-economic groups are less likely to be physically active during leisure time [53] and is likely to further reduce following a cancer diagnosis. It is known that there are inequalities in cancer survival by SES, with people from lower socio-economic groups more likely to delay seeking medical help after noticing symptoms [59]. In a study of the socio-economic inequalities in attitudes towards cancer, Quaife et al. [53] found that when SES was indexed by education, those with lower education levels reported significantly higher for the negative statements in the “Awareness and Beliefs about Cancer scale”; 57% of participants agreeing that “treatment is worse than cancer”, 27% that cancer is “a death sentence” and 16% “would not want to know if I have cancer”. This suggests that this group may be less receptive to promotional material for CARE and less likely to believe physical activity to be of benefit to them. Further work to understand the needs of this group in this context including barriers and facilitators to engaging interventions is therefore required.

When considering how PLWC were reached, focus group participants reported learning about CARE through health care professionals, including those in services where they had received their treatment, and this is similar to other studies [60]. It highlights the ongoing importance that health care professionals have in promoting physical activity to patients [61]. That said, there was variability and inconsistency with how people found out about CARE, with some participants finding out routinely through NHS services, while for others it was hit-and-miss and, in some cases, those motivated participants who undertook their own investigations on where to be active post-diagnosis uncovered CARE, sometimes through word of mouth. As such, some participants reported the need for more effective and routine promotion of CARE to patients by health care professionals. This may in part help to reduce the SES inequality noted previously.

In summary, these results suggest that in order for programmes such as CARE to reach PLWC who are currently under represented and arguably in most need of intervention, there is a need to engage PLWC more in their co-production [62], including strategies to promote physical activity programmes in this space.

### 4.2. Effectiveness

#### 4.2.1. Physical Activity

While the levels of physical activity seen in participants at baseline as measured by the SPAQ was generally high, with results suggesting that 95% of CARE participants exceeded current physical activity guidelines for health [22], this is inconsistent with what we normally see in the literature using other self-report [63,64] and objective measures [65]. We should therefore be cautious of the data provided by the SPAQ in this context, as respondents are likely to have overestimated their physical activity levels. While not reported in the methods, due to the perceived “failure”, there was an attempt by the authors to collect objective physical activity data on a sub group of CARE participants to supplement the SPAQ data, by training he programme manager to initiate and administer GENEActiv™ [66] accelerometers for 7 days at baseline, 3 and 6 months, but unfortunately this was not executed as intended and no data were collected. Despite the training and guidance provided, the programme manger reported that they lacked the confidence and capacity to implement the data collection, demonstrating that adequate resourcing and planning for intervention evaluations needs to be considered and secured before they begin [45].

Despite the high physical activity levels reported by the SPAQ, the same tool was used at each measurement period such that an indication of change can be made [38]. Results show that there was an overall significant increase in total physical activity measured by the SPAQ by 143 min per week at 3 months, which is in line with comparable studies using the SPAQ to measure physical activity [67]. Cancer treatment can have a considerable and prolonged negative impact on physical activity levels of PLWC, especially breast cancer survivors, leading to reductions in function and quality of life [63] and increasing the risk of comorbidities and recurrence [68]. Alongside the quantitative increase on physical activity, many focus group participants reported returning to physical activities and activity levels they enjoyed prior to diagnosis (Table 6) and given the barriers participants reported and without intervention, it is unlikely that the participants of CARE would have achieved this [63].

#### 4.2.2. Fatigue

Fatigue is the most common and distressing side effect of treatment reported by PLWC, which in turn has a negative impact on people’s wellbeing [7,8,9] which can persist for many years [69]. Indeed, for many PLWC, it is treatment related fatigue in particular that contributes to further reductions in physical activity [69]. For respondents in the present study, there was a significant improvement in fatigue scores at 3 months, which is coupled with the lived experiences of people reporting feeling less tired and attributing this to improvements in physical and psychological function.

#### 4.2.3. Health Related Quality of Life

Health related QoL is one of the important outcomes of treatment for PLWC and is associated with the multi-dimensions of wellbeing and life satisfaction which are negatively impacted after diagnosis [70]. Indeed, studies have shown that increased QoL is found to be predictive of survival time in breast cancer patients [71]. The results show that in line with the literature [5,7,8,9], CARE was successful in significantly improving their EQ-5D measured health related QoL over a three-month period. Of note is that participants reported an average score of 62.48 on the EQ-5D; mean values for the EQ-5D for people of this age are around 80 [72], suggesting that this sample rated their health considerable worse compared to population averages and indicating that intervention is warranted.

In relation to the EQ-5D dimensions (Table 8), while there were improvements in the proportion of participants reporting no problems walking, no pain or discomfort and not being anxious or depressed, there was also a proportion of participants that reported negative changes over the course of study period. Therefore, we need to treat these findings with caution, but it is likely that it represents the somewhat fluid nature of some participants’ health status (i.e., returning cancer) or the stage at which they entered the program. Their results might also suggest that such participants are able to engage with the program despite being unwell [38], supporting the literature that community delivered physical activity programs are feasible and safe for PLWC [73].

#### 4.2.4. Confidence in Daily Living

The General Self Efficacy questionnaire was included within the CaPASEF because of its validity and reliability and the association between increases in physical activity and confidence in daily living [38] and therefore to explore the assumption that an increase in physical activity and function would therefore translate into PLWC returning to activities of daily living. The average score for the Scale of 27.10 at baseline shows that this group of PLWC has a lower perception of confidence in daily life than international norm of 29.55 [74]. Despite the significant improvement over time, the mean score remained below the international norm at 3 months. Participants reported becoming efficacious when undertaking physical activity in the CARE program, in doing so becoming more technically proficient and feeling fitter to perform the frequency, intensity, time and type of physical activity. In some cases, participants reported being competitive against other participants especially male attendees in activities such as badminton and five-aside football. For some male participants competition is an important factor in facilitating adoption [32,33,34,35,36,37].

### 4.3. Adoption

In this study, participants reported that they had been active before cancer diagnosis yet following treatment many participants reported that their physical activity levels had decreased and even stopped. Higher pre-diagnosis and post-diagnosis physical activity levels have been found to be associated with improved survival outcomes for at least 11 cancer types [18]. It is encouraging that a number of participants reported the desire to take control, establish some form of normality in their life [36] and being physically active was part of this process. Yet, many participants reported facing barriers to the adoption of physical activity reported in the literature [75]. For most participants, CARE was able to alleviate these barriers, such as people’s fears and anxieties about being active and engaging “mainstream” exercise provision. Implementation characteristics that were influential in facilitating adoption by participants such as staff expertise, being in a similar position to other cancer survivors, venue and social support are discussed further in the Implementation section.

### 4.4. Implementation

Understanding perceived barriers and benefits to physical activity is a crucial step in designing effective interventions which facilitate adoption [76]. Persisting treatment-related side effects have been identified as the most commonly reported barrier to initiating or maintaining exercise [75]. In this study, participants referred to the physical effects of on-going treatment such as fatigue [60,76] and functional limitations from surgery, as well as their fitness levels and concerns surrounding their ability to perform exercises. Fears also extended to hair loss and weight gain and how they would feel or be perceived when attending a gym or a class. It is therefore unsurprising that participants reported concerns of engaging mainstream exercise provision and specifically the perceived awkwardness of having to explain the effects of cancer survivorship with instructors and participants alike hoping, but with little confidence that their concerns would be understood. However, an opportunity to be active with other people in similar circumstances can influence engagement in physical activity [60] and this featured in CARE. Catt and colleagues [60] have indicated that community-grown exercise initiatives bring cancer survivors together creating their own supportive environment. Participants referred to the social support they received from fellow attendees in the forms of encouragement (to stick with the exercise routines), interested enquiry (in the results of scans and treatments), competition (between participants, i.e., empathy and understanding), providing resources (such as shared transport and advice) and a general sense of togetherness at a challenging time.

Participants also expressed that there were a lot of negatives around cancer as reported in the literature [77], but “being active” was a positive [36]. In our study, respondents adopted a “glass half full” perspective, and in doing so providing accounts of people who were “worse off” then they were and so felt “inspired” to “persevere” with their rehabilitation. Further, many participants expressed wanting to take action and take control over their cancer [78] and physical activity and taking part in CARE was one way to achieve this [75].

Attendees expressed they had encountered a lack of expertise provided by staff in other mainstream physical activity services and expressed a desire to seek out appropriately qualified professionals who could help them become active. This was coupled with reported anxieties surrounding the frequency, intensity, time and type of activity, as well as concerns about developing an infection from the exercise settings [36], the impact of fatigue [79] and the risk of injury or exacerbating the severity of the effects of their treatment [80] including surgery. With CARE however, participants felt that staff not only knew attendees and their condition, but also helped reassure them that they would be both safe and supported when exercising under their care. This was only possible with staff who had the appropriate expertise and also an inclusive and person centred approach [81]. This highlights the importance of the staff who delivered the CARE program who were seen as skilled, trusted and knowledgeable and the relationship between practitioner and participant is one that developed over a period of time. As such, for those planning interventions, staffing and their expertise is an important implementation consideration, and this has been reflected in the literature on health improvement interventions delivered by football club community trusts [24,38].

### 4.5. Maintenance

In facilitating ongoing participation in the CARE program, attendees reported developing important self-management skills. Participants told us that attending CARE helped with scheduling a regular routine where attendees were physically active at the Portland Centre and then outside of CARE through informal and organised physically active events. Being physically active was therefore viewed as “a positive aspect”, when a lot of the experiences of cancer were viewed as negative [82]. The adoption of physical activity was also seen as “taking back control” of their lives and their conditions as reported in other long term conditions [83] and for some, this acted to motivate them to continue to engage in physical activity. This is important given the number of people who report being inactive, with less than half of cancer sufferers sufficiently active to benefit their health [14,15,16,17]. When at CARE, attendees also reported how exercises could be adapted around their functional limitations, participants also reported that developing the ability to evaluate the activities helped them to avoid overexerting themselves or get injured.; Being able to exercise proportionally was an important self-monitoring skill that was developed and examples like this point to how participants slowly built up their self-efficacy for participation in physical activity through the CARE environment, which is an important mediator long term physical activity participation [84].

Maintenance also relates to the continuation of programs [85], which was a sub-theme that emerged from the focus groups, as at the time, there was a concern about the future of the program following the end of the Macmillan funding. Securing the resources to maintain physical activity provision is an ongoing challenge, when it is considered there is a projected shortfall in the resources available for public health work by 2021 [86]. Since the completion of the Macmillan funding (June 2018) and as a result of the periodic reporting associated with this research, CARE has continued to be funded though the Nottingham City Clinical Commissioning Group and the East Midlands Cancer Alliance, putting CARE firmly in the Notts Cancer Pathway. NCF have continued the delivery of CARE as part of their provision in the City of Nottingham, but also further into Nottinghamshire, and across several sites beyond the Portland Centre. In March 2020, the emergence of COVID-19 as a global disease pandemic resulted in Government restrictions that necessitated the closure of the sport, fitness, and leisure sector, including the Portland Centre. However, during the two UK lockdowns designed to curtail the COVID-19 virus, flexible staffing at NCF enabled the CARE and other programmes to be delivered as an online offer to participants. This was undertaken using digital platforms where the exercise program was delivered by trained instructors but direct to people’s homes. In part, this reflects the efforts by community facing organisations including trusts and charities to help people during the unprecedented circumstances of COVID-19 [87]. The RE-AIM components of effectiveness and implementation of CARE as delivered using online platforms are subject to a future evaluation.

Another sub theme that emerged from the focus groups was “areas for improvement”, whereby participants voiced concerns about the ability of some staff to recognise the limits of PLWC. While the ability of staff to relate to PLWC and adapt exercise accordingly were important implementation and adoption sub-themes and domains, it is important to recognise that when up-scaling interventions such as this, that treatment fidelity may be compromised [88], such as due to core staff availability/illness. Ensuring that all people who deliver (or might deliver) specialised interventions such as CARE are trained to the appropriate level is essential. 

## 5. Strengths and Limitations

The main strength of this study is the mixed methods approach to evaluating a community-based intervention, providing in-depth participant accounts to better understand outcome level data. The researchers invested time to build up relationships with providers and participants before the project to facilitate data capture. By using the RE-AIM framework throughout the evaluation process, we were able to investigate notions of effectiveness and implementation identifying what works well and why, and what works less well and why. Further we were able to demonstrate the efficacy of providing cancer survivors with a community-based intervention that had a significant impact on their physical and mental function and provided commissioners and policy makers with rich information with which to base future applications and lobbying for resources. Indeed, the interim and final evaluation report was used to furnish the case to secure funding from Nottingham City Clinical Commissioning Group and the East Midlands Cancer Alliance to sustain the CARE program beyond the Macmillan funding period.

Gaining an accurate indication of the absolute reach of CARE was not possible, due to the broad eligibility criteria of the programme. The study was limited in part by the attrition of quantitative data to demonstrate long-term effectiveness and maintenance of change as a result of adopting CARE, but this is counterbalanced by the acquisition of the lived experience of people who have been attending the program for >12 months. As a result of limited funding available for evaluation, the present study had to rely on quantitative data collected across the 2 years prior, meaning that the authors could not inform the data collection process too much and was therefore hamstrung by the outcome measures used. Another limitation was the shift in program delivery part way though the outcome data collection period from a 12-week block approach to a rolling start pathway. It is therefore unclear as to whether this change had an impact on the results and therefore, we should be cautious. These factors may have contributed to the large between-subject variability observed, in addition to the likely impact of the varying cancers CARE participants presented with, which should also be treated with caution. In well-resourced and staffed interventions, the type of cancer and the treatment could be considered in the analysis of the CaPASEF questionnaire. With that said, this is the reality of evaluating real-world interventions and less rigorously controlled results should not be discounted. It is therefore important to acknowledge that conducting evaluative research in a community setting is complex and messy, with a multitude of uncontrollable variables that have an influence on the number of usable quantitative data, and which may not sit neatly within traditional experimental methodologies. That said, this approach to research is valuable and worthwhile as it provides a real-world view of the efficacy, acceptability and impact of interventions and services that are commissioned to improve population health outcomes.

## 6. Conclusions

The results of the present study demonstrate that CARE was effective in providing PLWC with a safe and supportive environment to adopt and maintain physical activity that had a positive impact on their physical (fatigue) and psychological (QoL) function, with some returning to levels they previously experienced before cancer diagnosis. While the significant questionnaire-measured physical activity changes across the three-month measurement period were likely to be over-inflated, this was likely due to the questionnaire used and the large between-subject variability, which may be as a result of the varied profile of the population’s stage of cancer diagnosis and treatment. The implementation of CARE including consideration for the components of people, place and process are important. Further research is needed into how interventions can best collect outcome data to ensure that they can effectively report their success (or otherwise). Engaging in mixed methods evaluations such as this are important for organisations such as football community trusts to be able to do just that and to justify future funding.

## Figures and Tables

**Table 1:** Components of the RE-AIM framework in the context of Cancer and Rehabilitation Exercise (CARE).

Construct	Definition as Applied in This Study	Data Source
Reach	The number, proportion, and representativeness of people living with cancer (PLWC) who participated in CARE.	Questionnaire data collected at baseline, 3, 6 and 12 months.
Effectiveness	The impact of CARE on physical activity, fatigue, health related quality of life, confidence in daily living.	Questionnaire data collected at baseline, 3, 6 and 12 months. Semi-structured focus groups with CARE participants.
Adoption	The profile of the PLWC who engaged in CARE including their physical activity, health status and reported barriers and facilitators.	Questionnaire data collected at baseline, 3, 6 and 12 months. Semi-structured focus groups with CARE participants.
Implementation	The key CARE intervention design and delivery characteristics that participants reported as being influential in facilitating their adoption.	Semi-structured focus groups with CARE participants.
Maintenance	The continued engagement of participants with CARE and the extent to which the intervention is sustained and can continue to be provided by Notts County Foundation.	Semi-structured focus groups with CARE participants.

**Table 2:** Summary of the of evaluation participants’ demographic profile at baseline.

Variable	Total (%)
**Gender (*n* = 169)**	
Male	48 (28%)
Female	121 (72%)
**Marital Status (*n* = 168)**	
Married/with partner	120 (71%)
Single/divorced/widow	48 (23%)
Other	10 (6%)
**Education (*n* = 165)**	
None	21 (13%)
General Certificate of Secondary Education or equivalent	42 (25%)
A level of equivalent	31 (19%)
Degree and above	71 (43%)
**Work Status (*n* = 165)**	
Paid work/Self-Employed	111 (6%)
Voluntary work	3 (2%)
At home/retired	45 (27%)
Student	1 (1%)
Other	5 (3%)
**Ethnicity (*n* = 163)**	
White	152 (93%)
Black/African/Caribbean/Black Asian/Asian British	9 (6%)
Other	2 (1%)
**Disability or illness (*n* = 159)**	
Yes	47 (31%)
No	109 (67%)
Prefer not to say	3 (2%)

**Table 3:** The number (%) of participants by cancer type, cancer status and treatment status at baseline.

Cancer Related Variables	Total (%)
**Cancer Type (*n* = 164)**	
Prostate	36 (22%)
Breast	91 (55%)
Colorectal	8 (5%)
Other	29 (18%)
**Cancer Status (*n* = 156)**	
Advanced or secondary metastatic	10 (6%)
Recurrence	3 (2%)
Stable	31 (20%)
Remission or cancer free	85 (55%)
Not known	23 (15%)
Other	4 (2%)
**Cancer Treatment (*n* = 159)**	
Treatment has not yet started	13 (8%)
I am currently in treatment	37 (23%)
The treatment has been effective	76 (48%)
Finished treatment but cancer still present	5 (3%)
Treated again, not fully responded to treatment	6 (4%)
Not in active treatment on ‘Watch and Wait’	17 (11%)
My cancer has not been treated	1 (1%)
Don’t know	4 (3%)

**Table 4:** Results of the paired samples t-test for the CaPASEF questionnaire components.

	Baseline		3 Months		Difference		*p* Value	95% CI
	Mean	(*SD*)	Mean	(*SD*)	Mean	(*SD*)		
Physical Activity (*n* = 68)	443.44	(251.52)	565.54	(221.62)	122.10	(261.92)	0.000 *	58.70–185.50
Fatigue (*n =* 89)	17.83	(11.30)	15.22	(13.31)	2.61	(10.71)	0.024 *	0.35–4.86
Quality of Life (*n* = 101)	62.48	(19.03)	73.86	(16.84)	11.39	(14.50)	0.001 *	0.51–3.45
General Self Efficacy (*n* = 90)	27.10	(4.50)	28.29	(4.35)	1.19	(3.27)	0.000 *	8.52–14.25

Note: * Denotes significant difference between baseline and 3 months.

**Table 5:** The number (%) of CARE respondents reporting baseline and 3 EQ-5D domains.

Dimension	Baseline	3 Months
**Mobility**		
No problems walking	17 (17%)	27 (26%)
Slight/moderate problems walking	34 (33%)	20 (19%)
Severe problems/unable to walk	52 (50%)	56 (54%)
**Self-Care**		
No problems washing or dressing self	19 (18%)	29 (28%)
Slight/moderate problems washing or dressing self	11 (11%)	8 (8%)
Severe problems/unable to wash or dress self	73 (71%)	66 (64%)
**Usual Activities**		
No problems doing usual activities	14 (14%)	21 (20%)
Slight/moderate problems doing usual activities	48 (47%)	38 (40%)
Severe problems/unable to do usual activities	41 (40%)	44 (20%)
**Pain/Discomfort**		
No pain or discomfort	14 (14%)	14 (14%)
Slight/moderate pain or discomfort	60 (58%)	55 (53%)
Severe/extreme pain or discomfort	29 (28%)	34 (33%)
**Anxiety/Depression**		
Not anxious or depressed	14 (14%)	22 (21%)
Slightly/moderately anxious or depressed	45 (44%)	31 (30%)
Severely/extremely anxious or depressed	44 (43%)	50 (49%)

**Table 6:** Overall Effectiveness themes, sub-themes and participant quotes related to their experience of CARE.

Theme	Sub-Theme	Quote
Functional Improvements	Physical Function	“I’m still here 28 months later. My posture has improved, my quality of life is so much better. I still can’t drive very far, but I can drive. I can actually run now, never mind walk. It’s turned my life around, so it’s been amazing for me. And, as somebody has said earlier, they thought I was a ballerina because my posture was so good. When I started, it really wasn’t, but it’s amazing what exercise can do if you stick at it.” (Female) “(It is) inspiring. Seeing where everyone’s come from and seeing how far everyone does through the weeks. You see some people who’ve just initially come, really struggling. You see them again in four weeks’ time, they’re doing the press-ups.” (Female)
	Psychological Function	“I had an intense five weeks; I didn’t exercise whatsoever, so I had to start again from scratch. So that’s since January, coming once a week. And I’m finding I’m getting a lot fitter, a lot better, feeling energised and more myself, sort of thing. ” (Female) “Well, it (cancer) made me feel like an old woman. I thought “this has made me an old woman before my time”. But when you do start to exercise, and you see yourself improving, it gives me more confidence and your mood, and your brain, I suppose, you feel a lot happier, you can see things happening, you can see yourself getting better, whereas at one point you just think it’s never going to happen.” (Female).
Improvements in Social Connectedness		“And I’ve started doing voluntary work myself, supporting people in a different environment. So it has begun to socialise me, I’ve begun to feel a bit more like I used to. ” (Female) “(At running club), I’m actually buddied up with a guy that had a liver transplant, so we’re both getting back to it, and he’s done me the world of good and I’ve done him the world of good, really. We just support each other, and we just take it steady. And it’s the best thing I’ve done, going back there twice a week. Yeah, it’s been absolutely fantastic. ” (Female)
Returning to “me”	Exercise	“I’m back running now at the gym. I’m running 5 or 6 miles. I did the Fever Challenge a couple of weeks ago, which was a run-walk. It was 15 miles. So, I’ve gone back to my running club, which has made me feel quite normal.” (Female). “It’s absolutely marvellous. It’s given me some of “me” back. We were discussing it this morning—I don’t think you ever get completely there, but it’s given me a lot of “me” back. (Instructor) is simply marvellous, and I don’t know what I’d do without him.” (Female)
	Work	“I was completely off work; I was signed off work for a year and a half. It made a huge difference, and I’m now back at work. Only two days a week.” (Male).

**Table 7:** Overall Adoption themes, sub-themes and participant quotes related to their experience of CARE.

Theme	Sub-Theme	Quote
Physical Activity Adoption	Previous Physical Activity Levels	“I was actually pretty fit before everything descended upon me. I was swimming about three mornings a week, doing a mile a time. So I think if I hadn’t been that fit I wouldn’t be here, because I’ve had cancer three times, and it does knock the stuffing out of you. I’m really a quite determined person, and I think I’ll just have to knuckle under.” (Male) “Somebody told me that I’ll start getting fat once I got to 50 and started the menopause, and I just thought ‘that’s not going to happen’. So that’s when I started. And somebody told me about Park Run. Well, I started that in 2014. That got me started running, and it’s absolutely fantastic. I’ve done about 110 now.” (Female) “I was diagnosed in November 2014 with breast cancer. Prior to that, I didn’t come to the gym or do any really conventional exercise. I like to do a little bit of swimming.” (Female)
Mental Wellbeing	Pre-CARE Mental Health	“There’s one lady who started (CARE)—I can’t remember her name. She was in tears, wasn’t she? So worried about going to do anything. We chatted to her and she ended up really enjoying it, didn’t she?” (Female) “What I would say is that from a confidence point of view, I’ve always been a very confident chap through my working life as a sales manager, I’ve always been very confident. So, the confidence as such has always been strong. But it would be fair to say that when I found I had cancer it did knock my confidence. I went through weeks of nightmares, “what’s going to happen to my family” and this sort of stuff.” (Male)
Barriers to Physical Activity	Fears and anxieties about being active	“For me, initially it was my surgery, I didn’t know […] Because I’d asked about getting into exercise and things, and they were saying ‘Oh, wait six weeks before you start any yoga or anything like that’. But I was just really concerned about what I could do and what I couldn’t do. I was reading all sorts of things online. Because I’d had a hysterectomy […] so women who had hysterectomies couldn’t get back into running […] Because I was quite into running as well, and that just put me off completely. I thought, ‘well, will I ever get back into it’?“ (Female) “If you’ve lost your hair, if you look ill, if you’ve put on weight! If you go to a class, you know—no disrespect to any teacher that is teaching it—you’ve got to then go ‘um, by the way…’ And you just feel […] Whereas with (the CARE Instructor), you don’t have to, you just have to say something if you’ve got a little problem or something like that. It’s lovely, and I think it’s well worth […] it’s real.” (Female)
	Loss of fitness and loss of an “active” identity	“Because that’s a part of it-exercise. If you’re really into something, that is part of your identity, yeah. You’ve lost something else. That you can actually get your fitness back. That’s exactly how I felt. It was a big frustration for me.” (Female) “When I came, there was one guy who could barely walk around the gym, there are people that are fighting, and that’s encouraging.” (Male)
	Effects of cancer treatment	“One of the problems for me […] I had chemotherapy both before and after the cancer. They tell me it takes about a year to get over the chemotherapy. Which I’d no idea […] if they’d told me exactly what they were going to do to me a year ago, I might not have bothered. They started off by saying ‘you’ve only got a 19 per cent chance of living’, and it goes on from there, really. ‘By the way, we have to break some ribs to get in from the back.’ So obviously your body’s a bit wrecked afterwards.” (Male) “I was kind of quite nervous about going (to CARE) […] to know what to do and what I could do. And, as I say, I didn’t want to go to a gym. I’d lost my hair; I’d lost my confidence.” (Female)
	Lack of social support	“I’d belonged to a gym, it’s very difficult to keep going to a gym when you’re going on your own, it’s very hard to motivate yourself. So, when this program was offered to me in 2015—that’s when I started, September 2015—I absolutely loved it. Also, because it got my mind off the treatments that I had. It was just a breath of fresh air, basically, to meet people who had suffered like myself or had the same kind of thing. It’s just nice to be able to meet this kind of people to talk about experiences.” (Female)
	Fears of engaging “mainstream” exercise provision	“You’re quite vulnerable, I think, especially when you first start on it […] until you get into doing the exercises. That’s why I didn’t want to go to a normal gym, because they all […] It is scary.” (Female) “You’re already trying to navigate your identity, after cancer. Trying to work out […] You don’t need everything else on top of it; you kind of just want people that understand you. You lose your identity, don’t you […] you just become the cancer patient, you know, like cervical cancer, breast cancer. And that’s what you get labelled with. It’s not intentionally, but I think you do lose it. it just wouldn’t be the same if we went to a normal exercise.” (Female)
Motives for Adopting CARE (PA)	Returning to physical activity participation.	“The breast nurse told me about the CARE program, and I came back. In fact, I came too early, I came two weeks after my reconstruction and they said I was a bit too early, so they sent me back for two weeks. (Laughs) They said, ‘come back in two weeks’. So, I’ve been coming since August. But I’m back running now at the gym. I’m running 5 or 6 miles.” (Female)
	Physical activity in a “safe” environment	“I just started to feel better, and I wanted to go to a gym. And the same problems that the ladies have said, like ‘I don’t want to be without my wig’. I mean, my hair’s grown back, but I still get cold and people look at you strange, don’t they? And I didn’t know whether I could actually do the exercise, if you know what I mean.” (Female) “I think that’s brilliant. To be honest, it actually pushed me to come more, because I’ve been to yoga and some of the exercise things that Maggie’s run. Seeing that it was Notts County, it’s a program outside of the cancer situation and maybe it’s someone that’s someone that’s qualified at what they’re doing.” (Female)
	Getting fit for surgery	“Having access for Portland, I did used to try and come once during the week to one of their classes. And I did lose that weight and felt much fitter and healthier going into that third surgery, which was great. Then I had the surgery and came back in the summer and have tried to come regularly ever since then.” (Female)
	Managing health conditions	“It is sharing the experience with other people, people are on a journey and as you move along the journey, people are at different stage, people are interested.” (Female). “It’s about restoration, actually coming and exercising, doing something for yourself, just gives you that little bit of yourself back. And that means that you’re then able to function better in the rest of the life and be there.” (Female)
	Distraction from medical treatment	“It (CARE) got my mind off the treatments that I had. It was just a breath of fresh air, basically, to meet people who had suffered like myself or had the same kind of thing. It’s just nice to be able to meet this kind of people to talk about experiences.” (Female) “It gives people a chance the really forget that they’ve got this Debilitating problem and put a smile on their face and again you can put a tick in a box with underlined on their faces and I hope that the program goes on for many years and grows because they need it.” (Male)
	Wanting on-going support and a positive approach	“So, by the end of the year you’re feeling pretty exhausted, although the treatment’s finished. But also, during that year I think you tend to be focused on your treatment and getting well. You’re going for loads and loads of hospital appointments, oncologists, radiotherapies, breast care nurses […] Then all of a sudden you get to the end of it, and it’s ‘Cheerio! We’re finished with you, off you go’!”. (Male Carer) “The main positive thing that’s probably come out of having the time off and having cancer is getting fit. I mean, I would never have gone to the gym before and done an exercise class. I always thought that’s for people who are really fit. I’ll just stick to swimming […] So yeah, around cancer it’s just always negative, you’re always told ‘you can’t do that, you can’t […] you’re going to lose your hair, this is going to happen to you.’ But actually, something positive […] You can come here and it’s such a positive atmosphere, even if you can’t do something.” (Female) “I can get the exercise anywhere. I can go to my gym on the Saturday and do that. But I can’t get this, and I can’t sit and talk to all the people who.” (Male)

**Table 8:** Overall implementation themes, sub-themes and participant quotes related to their experience of CARE.

Theme	Sub-Theme	Quote
Staffing	Staff skills, expertise and attributes	“The exercises are made fun and geared to all our needs. There’s no problem if you can’t do it the way it’s shown, the instructors find an alternative way for you. And we just laugh if we can’t manage it. They’re so encouraging and helpful.” (Female). “I think the CARE system is good, because it gets people who are not able, knowledgeable of what they are doing, or keen to do it, to actually do it. ‘Go do star jumps.’ What if you can’t do star jumps? Well there are three levels of star jumps. You can just do that if you want. So I think it works on so many levels […] I don’t think you could do that anywhere else. And then being in an open gym with all these fitness people doing what they’re doing and you’re just doing half a star jump. I think you’d be a bit self-conscious.” (Male)
	Participants trust the CARE staff	“They’re (the staff) very encouraging, all the staff. They’re just there and they’re helping you. You’ve got the exercises for each ability, so you know what you’re doing. And you can go up or down. And they’re there all the time, encouraging you—’oh, slow it down a little bit, you’re doing it a little…’ And you can trust them, you’re in their hands and you can trust them.” (Female)
	Responsive and enquiring staff	“I went to the “Moving On” course in January, that’s when CARE came along, and I said I was interested. And this kind of feeds into what you were saying about waiting lists […] because CARE staff phoned me almost immediately, I began to […] I came. If there had been a gap, I might have found plenty of reasons not to go.” (Female)
	Flexibility and Adaptability to Participant Needs	“I think that (the instructor), considering how many different types people he’s got, at different levels of fitness, what they can do and what they can’t do, I think he puts together a very structured program which has got something for everyone.” (Male) “On the side, he (the instructor)’ll talk to you. He has for some set personal goals to help with whatever our particular area of concern is. If you say, ‘what’s good about it?’ then I’m going to say, ‘All of it.’ I don’t mean that as a cop-out. It is just all so good.” (Male) “It’s handy because you know the free sessions are here, so you know you can drop in at any point. You know you’re not committed to saying ‘I’ll be here tomorrow morning.’ Because tomorrow morning I could feel rubbish and not be able to get here. But if I had 12 weeks and I knew ‘ok, I’ve got to really push myself and use these 12 weeks to sort myself out’, then it would be a totally new ballgame, wouldn’t it?” (Female)
	Providing a safe environment to exercise.	“I was quite sporty before, but I’ve not done anything in the past two years, so I was kind of quite nervous about going […] to know what to do and what I could do. And, as I say, I didn’t want to go to a gym. I’d lost my hair; I’d lost my confidence. Just having this group and knowing that everyone implicitly knows what you’re going through […] because we’ve got this whole thing that ties us together, it just made me so much more confident to go and exercise. So, I found that really, really beneficial.” (Female) “We’re not embarrassed to exercise. We do it by having people like us with normal people, you know. And they see you and they’re ‘Oh, actually that’s normal. It’s OK to do half a star jump.’ You don’t have to…you know. Because everyone’s different anyway.” (Male).
Social Support	Providing a supportive social environment	“There is a social element to what is achieved here as well. I mean, we’re all sort of becoming friends, I guess. We did a sponsored walk last year; we went bowling just after Christmas. It gives people a chance to really forget that they’ve got this debilitating problem and actually put a smile on their face. Again, that’s something you can’t put a tick in a box for, but it’s there, it’s underlying, and I think it’s extremely important. I hope the program goes on for many years and grows, because they really need it.” (Male)
	External social support from family members	“You know, my husband looks after our child on a Saturday morning so that I can come. It’s having that […] If I have that time, I’m better able to function, take care of the family, and go to work—because I’m fitter and healthier.” (Female) “My wife is a stronger person than me, she sort-of bats me around the head and things like that, if I don’t ‘get up and go’, so […] We’ve been married 41 years.” (Male)
	Reducing feelings of isolation	“Socially support environment also helps address the isolation that can be associated with the rehabilitation of long-term conditions. Because having cancer is, like you say, very isolating—your friends can be kind and all the rest, the same with partners, but nobody really experiences it as you do—so I stopped going out, I closed in. And this has started to bring me out. I now have an anchor to my week, a reason to come out. People in the group know what it is like to have cancer.” (Female)
Group Setting	Building trust and admiration within the group	“I think it’s managing a situation that is actually a form of treatment, which from a medical point of view, they make a diagnosis of treatment, and then they forget the years that people have got to live with the side-effects of the treatment. It means you’re not on your own. I must say, I admire a lot of people on this program.” (Male) “At our first meeting, it was ‘anyone was able to cry if they wanted to.’ And that really stumped me. I’d never been in with a bunch of blokes where that was something you could do. Actually, we’ve got something to give to other blokes, you can cry. I’ve done lots of crying. Blokes shouldn’t be afraid of crying. I think there are two types of people—those that are happy to talk about it and those that don’t. And I find in the social group that I’m in, I’m very happy to talk to anybody about it, and all my friends make jokes about it. But I know other people who are refusing to tell their wives, almost, about it.” (Male)
	Size of the group	“I particularly like it more now, because there are more people that are joining. And it’s great to get this new blood. In a way, I feel like I belong to a family. It’s become like my family.” (Female).
	Developing Empowerment and Positivity	“(CARE Staff are) really fantastic, they give you a power to lift up. That’s it. Like you’re coming back. Like a lot of things, after the cancer, you totally change your perspective on everything. Before this, you are really active, this and that. After, there’s a little bit of you lost.” (Female) “I think a lot surrounding cancer is like ‘you can’t do this…’ and everything around cancer is negative. And this is like the main positive thing that’s probably come out of having the time off and having cancer is getting fit. I mean, I would never have gone to the gym before and done an exercise class. I always thought that’s for people who are really fit. I’ll just stick to swimming. So yeah, around cancer it’s just always negative, you’re always told ‘you can’t do that, you can’t […] you’re going to lose your hair, this is going to happen to you.’ But actually, something positive […] You can come here and it’s such a positive atmosphere, even if you can’t do something.” (Female)
Developing Self-Management Skills for Exercise	Setting and working towards goals for improvement	“At the start they give some sort of measure of your fitness. I do appreciate for certain people that are not really for them. I think you’ve got great diversity within the group, and some people may not like that. But personally, I find that if I was given a target three months ago and found that I’d improved on it.” (Male) “I remember coming in one Friday morning, and the (instructor) actually spent 20 min with me, setting up a personal program which was relevant to my abilities. In other words, he said you’ve got to go on the rowing machine, and what I advise you to do is 2000 metres on the cycle. This was all written up as my personal training program which I could come and do on my own. But he gave me the first step.” (Male)
	Monitoring skills and techniques	“I think the best thing that (the Instructor) probably brought in, that I used to overdo at one time, is going into the red zone, pushing myself too much too quickly. He taught me that. It’s not a good thing to be in the red zone. And it’s not, really, when you think about.” (Female) “We have cancer, we’re normal people and we’re not embarrassed to exercise? We do it by having people like us with normal people, you know. And they see you and they’re “Oh, actually that’s normal. It’s OK to do half a star jump.” You don’t have to…you know? Because everyone’s different anyway. It was interesting when we did the rugby netball, basically a walking game where you throw the ball, but you’re not allowed to run and things like that. We still manage to push each other about, walking very quickly.” (Male)
	Establishing a routine for exercise	“I was completely off work; I was signed off work for a year and a half. It made a huge difference, and I’m now back at work. Only two days a week, still, but it made a huge difference to just have an arrangement where I had to be somewhere twice a week having a routine.” (Male)
	Learning the exercise skills	“We got different ideas and different bodies, whatever. But nobody actually said to me ‘while you’re doing that, you should be feeling a stretch there, or a stretch there.. And now (the instructor) says ‘when you stretch there, you’ll feel that…’ And now I understand what I’m stretching. Whereas before I was just stretching, thinking ‘well, I’m stretching, but…’ And then next time ‘Ooh, ah, that’s what it’s supposed to be doing.’” (Male)
	The location of the venue	“I think what has helped on the program […] the location for me is great, and I really admire people who travel so much further; because I’m not sure that I would have been committed to that.” (Female) “I travel from the North of the County, 45 min in traffic.” (Male)

**Table 9:** Overall maintenance themes, sub-themes and participant quotes related to their experience of CARE.

Theme	Sub-Theme	Quote
Areas for Improvement	Ability of all staff to understand participant limitations	“On a constructive criticism. I think sometimes, because all four of us in this room, to look at us we look fairly normal and we look well. And I think sometimes people (instructors) forget that we’re not well, and we do have problems, and I find occasionally people forget in class that you do have limitations. This is just a very minor point. I have to say ‘Hang on a minute, just remember I have got fatigue. Remember I can’t keep my arms up too long.’ People say, ‘Just hold your arms up and do it a bit more.’ No… no… And I think it’s great that we look really well, but I think sometimes people forget.” (Female)
	Standardised communications from staff	“Yes. I find it tends to be the assistants, not so much the senior staff. And it’s probably more so when I first started, but of course over time things have improved, if you see what I mean. Because (a) they know us, and they’ve got used to us. But if you get somebody new come into the class who is helping out—say an assistant who’s not so experienced or used to us, they forget, you know. I think one member of staff said to me ‘no pain, no gain.’ I said; ’In this class, that certainly doesn’t apply.’ I was very polite about it, but I don’t think that’s appropriate.” (Female)
	Promoting the CARE program	“I have always said that the posters do need to be a bit clearer. The word ‘cancer’ is nowhere […] well; it’s in the bottom in quite small print. They are around in the hospital. They’ve been there a while; they’re at the bottom of the stairs as you come down from chemotherapy. Mainly it’s […] half the poster is (the instructors) face, now I know it’s (the instructor), but the word ‘cancer’ isn’t; it’s just ‘CARE’. And I know […] we know what CARE is now, but I think the publicity of it could be slightly better.” (Female) “Maybe the posters need to be in different areas, like in the oncology unit or ward […] like in the chemotherapy places. While you’re sitting there waiting for treatment you do read them.” (Female) “Obviously, I was going for walks and things to build up my […] They encourage walking. For me as a young person—I suppose not even being young—as a person, exercise is part of life, isn’t it? So I’m really surprised that they didn’t recommend any groups or anything like that, because I did sort of ask, but […] again, especially like the nurses and things hadn’t heard of CARE either.” (Female)
Concern for the future of CARE	Duration of the program	“If you are re-diagnosed or if you do have further treatments, when can you access that 12 weeks, and at what point would you take on the 12 weeks? Because I started it during the end of my chemotherapy, but obviously I would question whether that was the right time, because I wouldn’t want to start it […] because I’d rather have it at the end […] if I knew that’s what I was having.” (Female) “It worries me, with it being a 12-week program; if that’s the way it’s going […] that obviously cancer doesn’t stop when you finish your treatment. And, as we’ve said, a few of us have had treatment after that. And it worried me that if I did have extra treatment then I wouldn’t have this program to come back to, to build up my strength again. I think that needs a lot of looking into to see what would happen in that case—whether people could enrol back on if they do have further treatment, or if that is it once […] Because I wouldn’t have the motivation to do all this without the group, so […] It does worry me.” (Female) “I think initially it was started for 12 weeks. I think, for people with cancer, 12 weeks is nothing. I’m talking about years. Which when I started off, I thought ‘Oh, press a button’ but the, drugs take 18 months to two years to wear off. They didn’t tell me that at the start. Post operation, lots of people I know, personal friends who have had the surgery, and they’re living with the side-effects for the rest of their lives.” (Male)

## Data Availability

The data presented in this study are available on request from the corresponding author. The data are not publicly available due to ethical restrictions.
